# Hemolysis in Pulsed Field Ablation for Atrial Fibrillation: A Narrative Review

**DOI:** 10.31083/RCM46485

**Published:** 2026-03-23

**Authors:** Angelica Cersosimo, Nicola Pierucci, Marco Valerio Mariani, Andrea Matteucci, Andrea Giovanni Parato, Vincenzo Mirco La Fazia, Andrea Natale

**Affiliations:** ^1^Department of Experimental and Clinical Medicine, “Magna Graecia” University, 88100 Catanzaro, Italy; ^2^Department of Cardiovascular, Respiratory, Nephrological, Anesthesiologic and Geriatric Sciences, Sapienza University, 00185 Rome, Italy; ^3^Division of Cardiology, San Filippo Neri Hospital, 00135 Rome, Italy; ^4^Department of Biomedicine and Prevention, “Tor Vergata” University of Rome, 00133 Rome, Italy; ^5^Texas Cardiac Arrhythmia Institute, St David’s Medical Center, Austin, TX 78705, USA; ^6^Department of Experimental Medicine, Tor Vergata University, 00133 Rome, Italy; ^7^Interventional Electrophysiology, Scripps Clinic, San Diego, CA 92121, USA; ^8^Case Western Reserve University School of Medicine, Cleveland, OH 44106, USA

**Keywords:** acute kidney injury, hemolysis, pulsed field ablation, atrial fibrillation

## Abstract

Pulsed field ablation (PFA) has emerged as a promising non-thermal energy source for treating atrial fibrillation (AF), demonstrating comparable efficacy to traditional thermal ablation techniques while offering an improved safety profile. However, recent evidence suggests that PFA may be associated with intravascular hemolysis, a complication that can potentially lead to acute kidney injury (AKI). This review aims to provide a comprehensive overview of the mechanisms of hemolysis associated with PFA and to summarize current strategies to mitigate the risk of AKI. The delivery of high-voltage electrical pulses during PFA can induce red blood cell lysis, resulting in elevated circulating free hemoglobin. The extent of hemolysis has been shown to correlate with several procedural variables, including peak output voltage, catheter-tissue contact quality, and, particularly, the number of energy applications delivered. Recent studies have highlighted that adequate preprocedural hydration may effectively reduce the incidence of AKI by promoting renal clearance of hemolytic products. Although hemolysis appears to be an unavoidable effect of pulsed field ablation, the clinical consequences associated with hemolysis, particularly AKI, can be significantly reduced with preventive measures.

## 1. Introduction

Catheter ablation for atrial fibrillation has conventionally been performed with thermal energy but is limited by the risk of collateral tissue injury [1, 2, 3, 4]. These safety concerns have led to the development of non-thermal approaches, particularly pulsed field ablation (PFA) [5]. This delivers ultra-rapid (nano and microseconds), high-voltage electrical pulses that induce irreversible electroporation, increasing membrane permeability and disrupting cellular homeostasis, ultimately leading to programmed cell death [6, 7, 8, 9, 10]. Different tissues have significantly different electroporation thresholds: cardiomyocytes have the lowest threshold among all tissues, requiring only 400 V/cm [11]. As opposite, nerve cells need 3800 V/cm [12], and vascular smooth muscle cells require 1750 V/cm [13].

Various devices utilizing PFA technology have been developed that could essentially be categorized into two groups: large footprint devices such as Farapulse (Boston Scientific), Pulseselect (Medtronic), Varipulse (Johnson&Johnson), and focal point-by-point devices such as Affera (Medtronic).

Large clinical studies have shown an association between pulsed field ablation and acute kidney injury related to procedure-induced hemolysis [14].

## 2. Hemolysis: Pathophysiological Mechanisms, Occurrence and Marker

Hemolysis refers to the destruction of red blood cells (RBCs), resulting in the release of free hemoglobin (Hb) into the circulation [15]. When this process occurs within the vascular system, it is termed intravascular hemolysis. Under normal physiological conditions, free Hb released into the bloodstream is rapidly bound by haptoglobin, a plasma protein that acts as a hemoglobin scavenger [15]. The resulting Hb-haptoglobin complex is then transported to the liver and spleen, where it is internalized and metabolized via macrophages expressing the CD163+ scavenger receptor. However, when hemolysis is excessive and the haptoglobin-binding capacity becomes saturated, free Hb remains unbound and circulates freely in the plasma [16]. In this scenario, the renal glomerulus becomes the primary route for Hb clearance. This filtration of free Hb can have nephrotoxic effects, particularly when the iron within ferrous hemoglobin (Fe^2+^) undergoes oxidation to form ferric hemoglobin (Fe^3+^), releasing free heme [16]. Free heme is a potent pro-oxidant and can trigger significant oxidative stress within the renal tubular epithelium. In detail, free heme and free hemosiderin, drive the majority of tubular oxidative stress, generating mitochondrial dysfunction, lipid peroxidation, and inflammatory injury. These pathways underlie hemoglobinuria-associated acute kidney injury (AKI) [17]. Importantly, the toxicity of free heme is not kidney-limited; oxidative injury may also affect the vascular endothelium, myocardial tissue, and reticuloendothelial organs, highlighting the systemic nature of intravascular hemolysis.

In cardiology intravascular hemolysis is observed particularly in patients using post mechanical circulatory support devices. This condition arises when mechanical stress on circulating red blood cells (RBCs) leads to their fragmentation and the release of cellular content into the bloodstream. Contributing factors to this phenomenon include inadequate preload and device thrombosis, both of which increase the mechanical stress on RBCs [18]. Therefore, the mechanism of PFA induced hemolysis is not completely understood but is suspected to be the exposure of red blood cells to high-voltage electrical pulses results in their lysis and subsequent release of free hemoglobin through a stepwise mechanism: the high voltage leads to leakage of ions, which causes colloidal hemolysis of the RBCs due to an induced osmotic imbalance [19].

Red blood cells begin to release their contents into the bloodstream when pulses of intensity ranging from 1100 to 1220 V/cm are applied. A significant percentage of red blood cells undergo lysis when the electroporation field reaches 1500 V/cm, and at levels exceeding 1600 V/cm, nearly all red blood cells are destroyed [20]. To achieve a lesion of 3 mm depth with optimal tissue-catheter contact, 700 V/cm is required. However, when the contact is suboptimal, achieving the same lesion depth necessitates increasing the energy up to 1500 V/cm [21]. Moreover, variability in catheter–tissue contact can increase the heterogeneity of the electric field and promote energy dispersion into the circulating blood pool [22]. Large-footprint or multielectrode catheters may accentuate this effect, especially in anatomically irregular regions such as the posterior wall and mitral isthmus, where stable contact is difficult to achieve. Biphasic waveforms, nanosecond–microsecond pulse durations, and higher peak voltages increase the degree of membrane permeabilization [23]. Repetitive pulse trains may further potentiate hemolysis by cumulative charge delivery [23]. Although PFA is designed to target tissue selectively, RBCs circulating in high-field regions remain vulnerable when exposed to suprathreshold intensities [24].

Considering the amount of applications required during the atrial fibrillation (AF) ablation in particular if applications beyond PV are required, becomes clear that hemolysis is almost unavoidable. The two single shot PFA devices, Farapulse and PulseSelect, release a bipolar pulse train with a maximum peak of 2000 V and 1600 V respectively [25]. The peak voltage of the PF waveform also contributes to the degree of hemolysis. In the presence of hemolysis, an increase in reticulocytes is expected as the bone marrow’s normal response to the peripheral loss of red blood cells [26]. An increase in unconjugated bilirubin and LDH and a decrease in haptoglobin levels accompany the destruction of red blood cells. Then once haptoglobin’s capacity to bind free hemoglobin is saturated, the free hemoglobin is filtered by the glomerulus, leading to hemoglobinuria. This condition causes red-brown urine and is indicated by a positive urine test for heme in the absence of red blood cells [27].

## 3. Current Evidence

The current literature provides limited but growing insight into the relationship between hemolysis and AKI following PFA. Although PFA has been widely recognized for its myocardial selectivity and favorable safety profile, emerging data indicate that intravascular hemolysis is a consistent occurrence during the procedure and may, in some circumstances, contribute to renal injury.

The first report was provided by Venier *et al*. [28], who studied 68 consecutive patients undergoing AF PFA with the Farapulse system between June and October 2023. In this cohort, 28% of patients demonstrated laboratory evidence of hemolysis, as evidenced by a significant reduction in haptoglobin levels below 0.04 g/L. Additionally, 27% of patients showed signs of hemoglobinuria, 77% had elevated lactate dehydrogenase (LDH) levels, and 35% exhibited increased levels of free hemoglobin [28]. Despite these laboratory abnormalities indicating red blood cell lysis, no cases of AKI were observed post-procedure in this specific cohort. However, the same group reported two additional cases of AKI due to intravascular hemolysis following PFA with the Farapulse system, which occurred in May and June 2023 [28].

In another study by Mohanty *et al*. [22], 28 patients underwent PFA, and 21 (75%) developed hemoglobinuria. Four of these patients progressed to AKI, reinforcing the potential renal implications of extensive hemolysis. Both the Mohanty *et al*. [22] and Venier *et al*. [28] studies clearly demonstrated a positive correlation between the number of PFA applications and hemolysis severity. Patients with hemoglobinuria had significantly higher LDH and total bilirubin levels, along with lower haptoglobin concentrations. In the first study [28], patients who developed hemoglobinuria had received an average of 57.38 ± 22.78 PFA applications compared to 32.43 ± 4.27 in those who did not. Among the four patients who developed AKI, the number of applications was notably higher. Similarly, in the second study [22], patients with hemoglobinuria received between 62 and 127 applications, while those without hemoglobinuria had between 40 and 60. The two patients who developed AKI had undergone 174 and 126 applications, respectively. Furthermore, a positive correlation has been reported between the number of PFA applications and the concentration of RBCs [29], which is indicative of hemolysis.

Recent evidence suggests that the degree of hemolysis associated with PFA may vary depending significantly on the type and size of the ablation catheter used [30]. In particular, larger, multi-electrode catheters appear to be linked with a higher incidence of hemolysis compared to smaller, point-by-point catheters. This was highlighted in the NEMESIS-PFA study [31], which systematically investigated tissue injury and hemolysis across different catheter platforms used in PFA procedures. In detail, these authors reported that patients treated with larger lattice-style or multi-electrode catheters demonstrated elevated levels of hemolysis markers, including lactate dehydrogenase (LDH) and plasma-free hemoglobin, when compared to those treated with smaller, focal-tip catheters [31]. The difference in hemolysis burden may be attributable to catheter-tissue interface characteristics. Larger catheters, while designed for anatomical efficiency and faster pulmonary vein isolation, may not achieve consistent wall contact in anatomically complex regions such as the posterior wall or ridge between the left pulmonary veins. In these regions, incomplete or unstable contact can result in unintended energy dispersion into the circulating blood pool rather than focused delivery to myocardial tissue. Indeed, dispersion increases the likelihood of red blood cell damage due to the electroporation effect extending into the blood rather than being confined to the tissue [32]. A recent study by Martinez *et al*. [33] found a correlation between the number of PFA applications and creatinine increase, estimating 129–140 applications to observe a 0.3 mg/dL rise, while no association was observed between hemolysis markers and AKI. Importantly, even patients receiving high volumes and high numbers of PFA pulses without pre-procedural saline loading did not experience significant renal deterioration.

These findings collectively underscore that the number of PFA applications is a key determinant of hemolysis severity and the associated risk of AKI. Based on the current evidence, a threshold of approximately 100 applications may represent a critical cut-off for identifying patients at higher risk for renal complications. Importantly, patients with pre-existing renal impairment may develop AKI even with a lower number of applications, highlighting the need for tailored procedural strategies, careful monitoring, and the use of pre-procedural hydration to mitigate renal risk. Table 1 (Ref. [27, 29, 30, 31, 34, 35]) summarizes the current evidence about hemolysis during PFA procedure.

**Table 1.** **Characteristics of PFA procedures and associated risk of hemolysis-induced AKI**.

Study	Population	Ablation strategy	AKI occurrence	Hemolysis markers	Conclusions
Auf der Heiden *et al*. [34]	200	PFA vs RFA	No	LDH, bilirubin, urea	PFA showed no safety concerns with respect to hemolysis-induced AKI
Jordan *et al*. [35]	2570	PFA vs RFA vs cryo	3.3%	LDH, bilirubin, urea	AKI is rare when PFA is used in a standardized fashion with no extensive high number of applications and is lower than that after thermal ablation
Kawamura *et al*. [30]	90	PFA (pentaspline vs circular multielectrode catheter) vs cryoablation	No	LDH, bilirubin, haptoglobin	Farapulse had significantly higher levels of hemolysis than PulseSelect
Lakkireddy *et al*. [31]	871	PFA (pentaspline vs circular multielectrode, vs spherical vs variable loop catheter)		LDH, troponin, haptoglobin	PFA was associated with hemolysis
Osmancik *et al*. [29]	70	PFA vs RFA	No	RBCµ, LDH, bilirubin, haptoglobin	Hemolysis occurred during PFA of AF, far exceeding what occurs during RFA. The extent of hemolysis depends on the number of PF applications
Popa *et al*. [27]	215	PFA vs RFA	3.2%	Haptoglobin, LDH, bilirubin	Intravascular hemolysis is a frequent finding after PFA and increases with the number of PFA deliveries

Abbreviations: AKI, acute kidney injury; LDH, lactate dehydrogenase; PFA, pulsed-field ablation; RFA, radiofrequency ablation; RBCµ, red blood cell microparticles; AF, atrial fibrillation; PF, pulsed field.

## 4. Hemolysis in Thermal Energy Sources

With thermal energy sources severe renal dysfunction as a complication is rare and mostly anecdotal [36]. The available data comparing the impact of hemolysis in AF ablation procedures using thermal sources (radiofrequency (RF) and cryoablation) and non-thermal sources (PFA) are limited and somewhat conflicting.

Modest alterations in hemolysis markers, specifically, only borderline changes in LDH and haptoglobin, and a mild increase in RBC microparticles (RBCµ) are observed following RF ablation, although these changes appear less pronounced than those associated with PFA. Additionally, no significant variation in serum creatinine concentration is detected after RF ablation. This limited degree of hemolysis is likely attributable both to the direct thermal effects of RF application (i.e., red blood cells *in vitro* undergo budding and fragmentation at temperatures above 49 °C) and to indirect effects mediated by coagulation activation, which generates mechanical stress [29]. A comparative study investigating the occurrence of hemolysis in patients undergoing PFA versus radiofrequency (RF) or cryoballoon ablation reported conflicting results [35]. The authors observed that acute AKI occurred less frequently in the PFA group, and fewer patients showed altered markers of intravascular hemolysis compared to those treated with thermal energy sources. However, significant differences in group sizes may have influenced the findings: 1707 patients in the RF group, 557 in the cryoballoon group, and only 306 in the PFA group. Additionally, the average number of applications per patient was higher in the PFA cohort (32 applications), potentially introducing procedural variability [35]. The hemolysis markers used (lactate dehydrogenase and bilirubin) are nonspecific and may not accurately reflect the true incidence of hemolysis. Moreover, hypotension during general anesthesia, a known contributor to renal injury, was not uniformly controlled for and could have confounded the results. These factors collectively raise concerns about potential misinterpretation of the extent of hemolysis among the different treatment groups [24].

## 5. How to Minimize the Risk of AKI

Hemolysis is an almost inevitable consequence of PFA procedures for AF. While this phenomenon cannot be entirely prevented due to the intrinsic nature of the energy delivery mechanism, its physiological repercussions. Particularly, its impact on renal function, must be carefully managed. The breakdown of red blood cells releases free hemoglobin and other intracellular contents into the circulation, which, if not promptly cleared, can accumulate in the renal tubules and contribute to AKI. Therefore, strategies aimed at mitigating the renal impact of hemolysis are crucial, especially in patients undergoing a high number of applications during the procedure.

One of the most effective and straightforward approaches to protecting renal function is the implementation of an adequate hydration protocol before and during the procedure. Pre-procedural intravenous administration of fluids has been shown to play a protective role by enhancing renal perfusion and diluting circulating hemoglobin and cellular debris.

The high-voltage electrical pulses cause hemolysis, releasing free hemoglobin into the bloodstream. Hemoglobin-induced tubular-barrier deregulation and oxidative cell damage result in AKI.

**Fig. 1.**
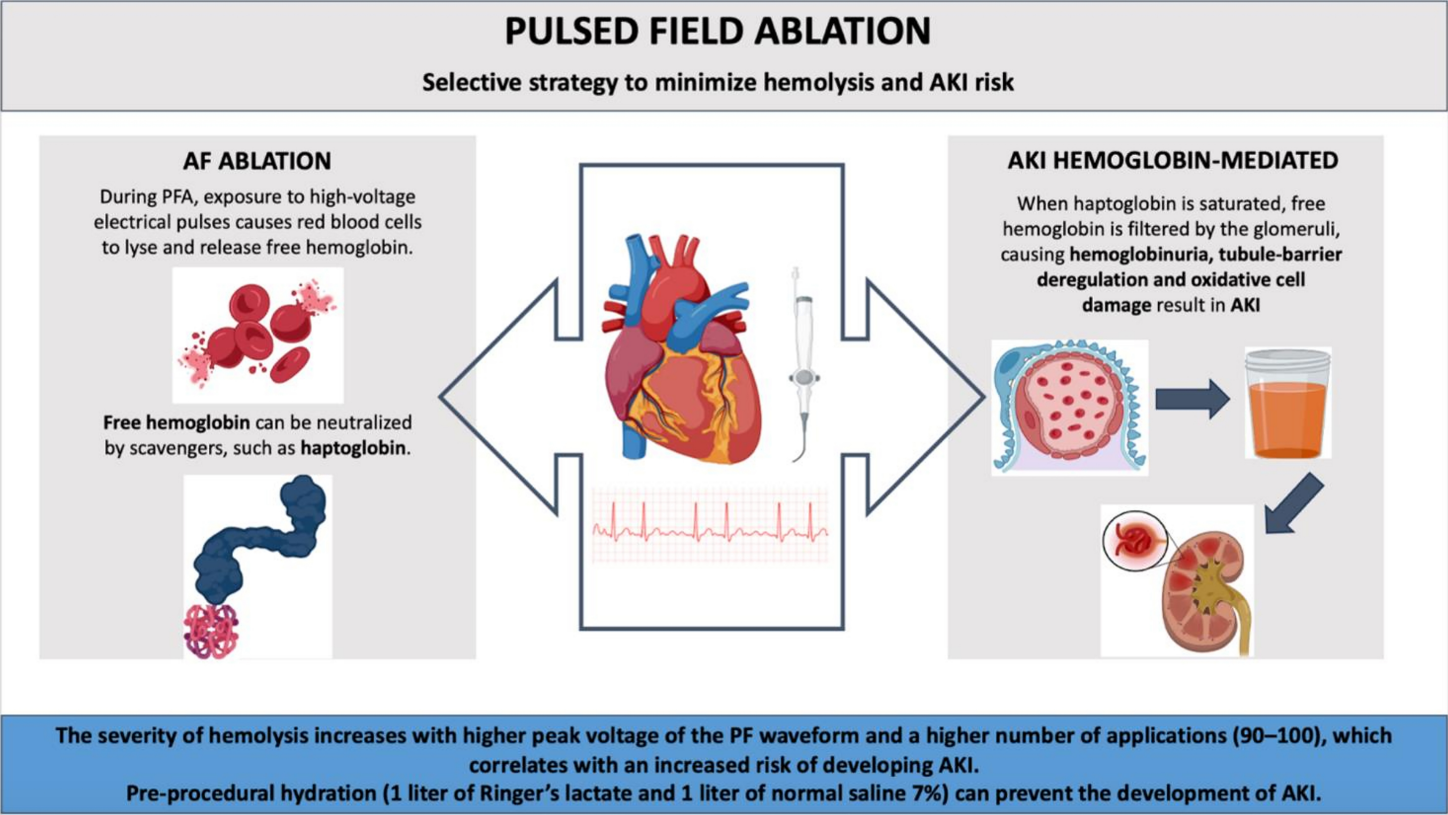
**The mechanism and prevention of acute kidney injury during atrial fibrillation pulsed field ablation**. Abbreviations: AF, Atrial Fibrillation; PFA, Pulsed field ablation; AKI, Acute Kidney Injury. Created with Biorender.

The severity of hemolysis increases with higher peak voltage and a higher number of applications (90–100), correlating with an increased risk of AKI. Free hemoglobin can be neutralized by haptoglobin. However, when the capacity to bind free hemoglobin is exceeded, it is filtered by the glomerulus, leading to hemoglobinuria. Given the potential for hemolysis to cause downstream complications, including AKI, strategies to minimize this risk are essential (Fig. 1).

Adequate periprocedural hydration is a key preventive strategy to reduce the risk of acute kidney injury after pulsed field ablation. Pre-procedural intravenous fluid administration has been shown to lower AKI incidence by improving renal perfusion and facilitating clearance of hemolysis-related byproducts [37]. However, signs of hemolysis have been reported even in hydrated patients. Therefore, hydration should be considered part of a broader risk-mitigation for AKI.

In addition to maintaining adequate hydration, it is essential to avoid periprocedural hypotension, which represents another important and potentially preventable risk factor for renal injury [38]. Hypotension can compromise renal perfusion, exacerbating the effects of hemolysis [39]. One common cause of periprocedural hypotension in AF ablation is a vagal response, particularly during energy delivery near the left superior pulmonary vein. To prevent this, administration of 1 mg of atropine after successful transseptal access is recommended. Atropine, an anticholinergic agent, effectively blunts the parasympathetic response, thus reducing the likelihood of vagally mediated bradycardia and hypotension during ablation [40].

Furthermore, ensuring optimal catheter-tissue contact is critical in maximizing the efficiency of energy delivery and minimizing the unintended release of energy into the blood pool, which can increase hemolysis. Applications delivered with poor contact may result in excessive interaction between the pulsed field energy and circulating blood, leading to higher levels of red blood cell destruction. To avoid this, operators should aim to confirm stable and effective contact between the ablation catheter and the targeted myocardial tissue. Intracardiac echocardiography enables real-time visualization of catheter position and stability, allowing operators to adjust and confirm proper apposition before energy delivery. This minimizes applications in regions with suboptimal contact, where energy could otherwise be directed into the bloodstream, increasing the risk of hemolysis. For instance, Mohanty *et al*. [41] recently showed that the use of ICE significantly improves the quality of contact between the ablation catheter and atrial tissue.

**Fig. 2.**
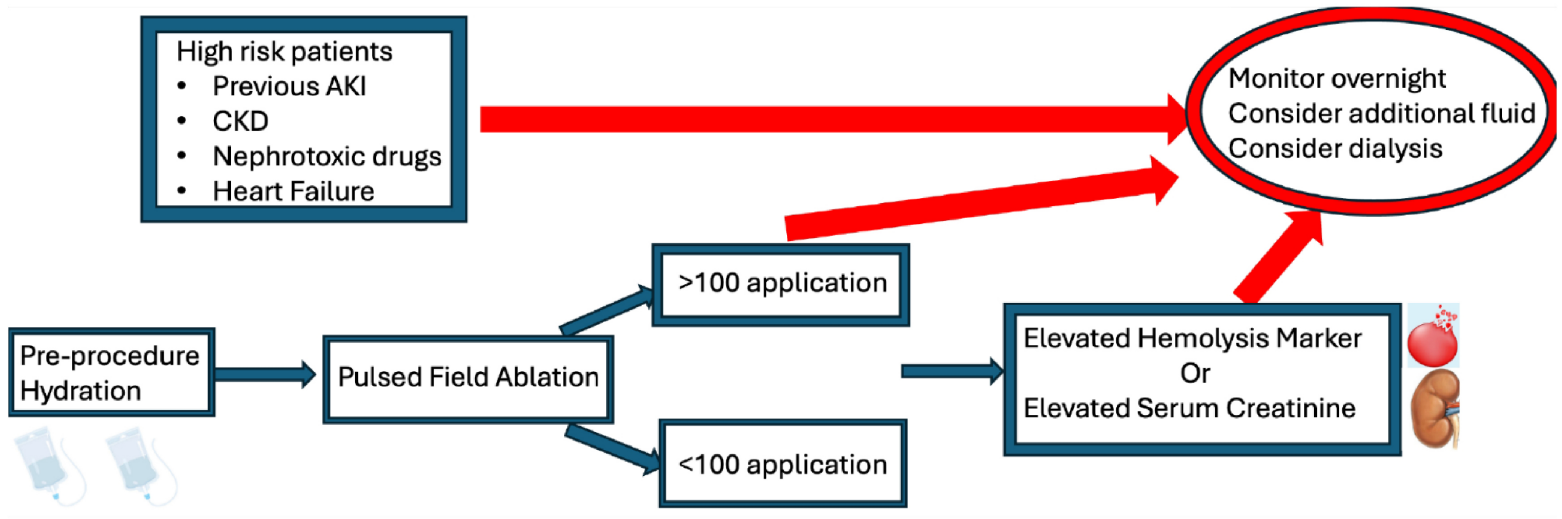
**Flow chart for preventive strategy to reduce acute kidney injury**. Abbreviations: CKD, chronic kidney disease; AKI, Acute Kidney Injury. Created with Biorender.

Although hemolysis during AF PFA is largely unavoidable, its downstream effects, especially on renal function, can be significantly attenuated through a combination of proactive hydration, hemodynamic stability, and careful procedural technique. These measures are essential in optimizing patient outcomes and reducing the incidence of post-procedural AKI (Fig. 2).

## 6. Hemolysis Across Different PFA Systems

Available evidence indicates that hemolysis is a frequent but heterogeneous phenomenon following PFA, with substantial variability related both to the methods used for its assessment and to the catheter technologies under investigation. Kawamura *et al*. [30] evaluated hemolysis using a panel of biochemical markers, including free hemoglobin, LDH, total bilirubin, and haptoglobin, measured before and after the procedure, demonstrating significant differences between single-shot PFA systems despite a comparable number of applications. Their findings suggested that system-specific characteristics contribute to hemolytic burden, while the absence of acute kidney injury supported a predominantly subclinical manifestation of hemolysis.

Gianni *et al*. [42] conducted a large retrospective analysis that systematically evaluated the extent of hemolysis associated with different PFA technologies. In this study, haptoglobin was used as the primary marker of intravascular hemolysis, complemented by free hemoglobin, LDH, indirect bilirubin, and renal function parameters, allowing for a comprehensive biological and clinical characterization [42]. Although a reduction in haptoglobin was observed in the vast majority of cases, the severity of hemolysis differed markedly across catheter types. Notably, the focal Sphere-9 catheter, characterized by a smaller size allowing better contact, was associated with significantly lower rates of significant and severe hemolysis compared with larger single-shot catheters such as Farawave, PulseSelect, and Varipulse. These findings suggest that catheter geometry and size may play a key role in limiting erythrocyte exposure to the electric field, beyond the effects of application number or nominal voltage.

Consistent results were reported by Bruss *et al*. [37], who compared all PFA platforms commercially available in Europe using haptoglobin and LDH as markers of hemolysis. Their analysis confirmed that focal lattice-tip catheters induced a significantly smaller decrease in haptoglobin both per procedure and per application than larger footprint catheters, further supporting the relevance of catheter design in modulating hemolytic effects.

Taken together, these studies indicate that PFA-related hemolysis is strongly influenced by system-specific factors. In particular, available evidence highlights that smaller focal catheters are associated with a more favorable hemolytic profile, a finding with potential implications for tailoring ablation strategies in patients at increased risk of hemolysis-related complications.

## 7. Conclusion

Exposing red blood cells to high-voltage pulses during PFA inevitably leads to hemolysis. With a high number of applications (90–100), the severity of hemolysis increases, correlating with a higher risk of developing AKI. A strategy to minimizes the risk of AKI includes limiting applications to only those essential for therapeutic goals and ensuring adequate hydration before the procedure.
